# Species-specific traits determine halophyte nutrient patterns rather than plant functional types

**DOI:** 10.1038/s41598-026-49735-1

**Published:** 2026-04-21

**Authors:** Pedro Valle-Romero, Elena Romano-Rodríguez, Enrique Mateos-Naranjo, Susana Redondo-Gómez

**Affiliations:** Departamento de Biología Vegetal y Ecología, Facultad de Biología, Universidad de Sevilla, Avda. Reina Mercedes s/n, Seville, 41012 Spain

**Keywords:** Biota, Functional type, Hydrohalophyte, Physical-chemical characteristics, Soil, Xerohalophyte, Ecology, Ecology, Environmental sciences, Microbiology, Plant sciences

## Abstract

Halophytes are plants capable of completing their life cycle in saline soils and, depending on their ecological strategies and the environmental conditions in which they grow, they may be broadly classified into two plant functional types (PFTs): hydrohalophytes and xerohalophytes. We investigated whether distinct nutritional patterns were associated with these PFTs by comparing the nutrient content of three hydrohalophyte and three xerohalophyte species in relation to soil physicochemical properties, microbial communities, and enzymatic activities. Our results suggest that halophyte nutritional status was related to species-specific traits and local soil conditions, rather than being explained solely by PFT classification. For instance, hydrohalophytes *Atriplex portulacoides* and *Salicornia perennis* exhibited similar nutritional profiles despite contrasting soil physicochemical conditions. Statistical analyses identified soil pH, electrical conductivity, organic carbon, and clay and silt contents as key environmental factors associated with variation in plant nutrient concentrations. Moreover, a positive correlation was observed between silt and clay textures and microbial biomass, indicating a potential role for soil-microbe interactions in nutrient cycling. Overall, these findings indicate that nutrient dynamics in halophytes may not be fully predicted by PFT categories alone, highlighting the complex interplay between species traits and site-specific soil conditions. This underscores the value of mechanistic, species-level studies for improving our understanding of nutrient acquisition and cycling in saline ecosystems.

**Supplementary Information:**

The online version contains supplementary material available at 10.1038/s41598-026-49735-1.

## Introduction

Halophytes are salt-tolerant plants that can complete their life cycle under extreme salinity in environments such as coastal salt marshes and saline deserts, where they are subjected to high environmental pressures^1,2^. According to the environmental conditions in which they grow, halophytes are further divided into two plant functional types (PFTs): hydrohalophytes and xerohalophytes^3,4^. Hydrohalophytes are found in aquatic conditions, or wet soils such as mangroves and salt-marsh species inland or along coastlines. Xerohalophytes survive in dry habitats with saline soil, occurring in arid and semi-arid inlands with unpredictable rainfall^5,6^. Whatever the case, when they grow and reproduce under extreme salinity conditions, i.e. $\:\ge\:$ 500 mM NaCl, they are regarded as extremophiles^7^. Halophytes show anatomical, morphological and physiological adaptations that enable them to survive in these highly stressful environments, reflecting not only their potential for salt tolerance but also their efficiency in using water and nutrients^8–10^.

Several studies have demonstrated that the nutrient content of plants is related to the content of the soil in which they grow^11,12^. Therefore, the presence of mineral nutrients in the growing environment along with the ability of the plant to absorb specific mineral nutrients will determine the nutritional status of plant^13^.

Soil properties, including texture, electrical conductivity (EC), pH and organic matter content, strongly influence the amounts and availability of mineral nutrients in soil^12,14^. Thus, for example, soil salinity has a detrimental effect on nutrient absorption by plants^15^. In arid and semi-arid environments, where high evaporation and limited rainfall intensify salt accumulation, salinity and alkalinity are key drivers shaping soil physicochemical conditions^16^. In this sense, dry saline soils, associated with xerohalophytes, often exhibit alkaline conditions, elevated salinity and water limitation. In contrast, wet saline soils, typically found in hydrohalophyte habitats, tend to have near-neutral pH, higher moisture and greater organic matter. These differences likely result in distinct microbial communities, enzymatic activities and nutrient cycling dynamics in each habitat^17^. Additionally, soil biota, particularly plant growth-promoting bacteria and arbuscular mycorrhizae, have been reported to play a key role in facilitating the uptake of mineral nutrients^18,19^. Consequently, the nutritional status of the plants will be influenced by these interactions^12^. In turn, soil microbial communities also affect the enzymes that regulate nutrient circulation in soils^20,21^. Phosphatase, in particular, plays a critical role in nutrient cycling and soil health and is highly sensitive to changes in the soil environment^22^. In this way, irrigation has been described as strongly enhancing the activities of alkaline phosphatase, dehydrogenase, β-glucosidase, urease and arylsulphatase in the soil^23^. Anyway, the enzymatic activity of rhizospheric soils is greater than that of bare soil because plant root exudates stimulate microbial activity^24^. Finally, Mora-Ruiz et al.^25^ found that soil physicochemical properties affected bacterial community composition associated with the halophyte *Arthrocnemum macrostachyum*. Thus, nutrient uptake by plants reflects the integrated effects of soil properties, microbial communities and enzymatic activities. Comparing these habitats is essential to understand how variation among species in interactions with soil and microbes is associated with nutrient status beyond PFTs.

Nonetheless, the relationship between mineral nutrient concentrations in the soil and in plant tissues is highly variable and dependent on the individual nutrient and its role in plant metabolism^26^. This variability is driven by interspecific differences in nutrient acquisition and utilization strategies, which are mediated by molecular mechanisms such as the regulation of transporter genes, signaling pathways and metabolite production that modulate nutrient uptake and assimilation^12,27^. Therefore, plant nutrient acquisition, utilization and metabolism are influenced by intricate interactions among plants, soil and microorganisms, along with the genetic and molecular mechanisms that govern plant responses to environmental stresses and nutrient shortages^28–30^.

Considering the background described above, we hypothesize that there will be a specific nutritional pattern of halophytes according to the ecological strategy they present, that is, whether they are hydro- or xero-halophytes. Furthermore, this pattern is expected to depend on the variations in enzymatic activities developed by the soil microbiota, which in turn will depend on soil physicochemical properties. To our knowledge, no previous study has been conducted addressing this issue. Most studies focus on analyzing the nutritional value of halophytes as alternative crops^31,32^ and some which study C:N:P stoichiometry of different halophytes to gain deeper insight into how halophytes adapt to coastal environments^10^. In summary, this study aimed to compare the nutritional content of three species of hydrohalophytes and three species of xerohalophytes, each of them distributed across different biogeographical regions of the Iberian Peninsula, in relation to soil physicochemical properties, microbiota composition and soil enzymatic activities. This study also evaluates which of these factors is most decisive in explaining the nutritional status of each functional type of halophytes.

## Materials and methods

### Sample collection

#### Study species

The hydrohalophytes selected were the shrubby species *Atriplex portulacoides* (L.), *Suaeda vera* Forssk. ex J.F. Gmel., and *Salicornia perennis* (Mill.). These three species are found in the Atlantic coastal ecosystems of the Iberian Peninsula with tidal influence or with wet soil, where they have developed well established populations, mainly in tidal marshes and in inland salt lakes^33^. The xerohalophytes selected were *Atriplex glauca* L., *Anabasis articulata* (Forssk.) Moq., and *Halocnemum strobilaceum* (Pall.) M. Bieb., all of which are found in dry habitats with saline soils, characteristics found in Mediterranean coastal areas from the Southeast arid extreme of the Iberian Peninsula^33^. All six species are perennial obligate halophytes belonging to the Amaranthaceae family and share an emergent growth habit. They were selected as representative species of each plant functional type to compare nutrient patterns at the species level under contrasting saline environments while minimizing differences in life history traits. Moreover, these species have been previously used in comparative studies addressing plant-microbiome interactions in hydro- and xerohalophytes, providing a well-established ecological and microbiological framework for their selection^17,34^. Identification of the plant material was carried out in collaboration with the Herbarium Service of the University of Seville (CITIUS), according to Castroviejo et al.^33^. Voucher specimens have been deposited there, with accession numbers provided in Table 1.

**Table 1:** Sampling coordinates and Herbarium Service (CITIUS, University of Seville) deposition numbers from each population of the studied hydro- and xerohalophyte species.

Functional type	Species	Population name	Coordinates	Herbarium deposition No.
Hydrohalophytes	*Atriplex portulacoides* *Salicornia perennis* *Suaeda vera*	Duque	37°11’27.5"N 7°20’47.5"W	290909
				290910
				290911
	*Atriplex portulacoides* *Salicornia perennis* *Suaeda vera*	Estadio	37°12’13.1"N 7°24’28.0"W	290912
				290913
				290914
	*Atriplex portulacoides* *Salicornia perennis* *Suaeda vera*	Terrón	37°13’20.8"N 7°10’41.0"W	290915
				290916
				290917
Xerohalophytes	*Atriplex glauca*	Ermita	36°49’09.2"N 2°17’19.5"W	290918
	*Atriplex glauca*	Rambla	36°43’30.9"N 2°11’36.8"W	290919
	*Atriplex glauca*	Corralete	36°43’34.0"N 2°11’42.0"W	290920
	*Anabasis articulata*	Fabriquilla	36°44’12.9"N 2°12’20.4"W	290921
	*Anabasis articulata*	Faro	36°43’20.6"N 2°11’29.1"W	290922
	*Anabasis articulata*	Isleta	36°46’39.3"N 2°03’34.9"W	290923
	*Halocnemum strobilaceum*	Guadalentín	37°48’47.8"N 1°25’09.8"W	290924
	*Halocnemum strobilaceum*	Calarreona	37°23’06.5"N 1°37’23.4"W	290925
	*Halocnemum strobilaceum*	Ecoparque	37°49’24.7"N 1°23’55.0"W	290926

Voucher numbers correspond to specimens identified and deposited at the Herbarium of the University of Seville (CITIUS).

#### Sampling areas

The hydrohalophytes were sampled in marshes of the Gulf of Cadiz (SW, Spain). The Mediterranean climate in this region is characterized by a strong seasonality, with dry and hot summers, and mild wet winters. These salt marshes constitute a natural mesotidal enclave of fluvial-tidal modeling and coastal feeding. The tides occur twice daily, with an average range of 2.10 m and an average spring tidal range of 2.97 m, varying between 0.40 and 3.37 m above the Spanish Hydrographic Zero^35^. In each of the three marshes, a population of each of the hydrohalophyte species was sampled (Table 1).

The xerohalophytes were sampled in independent plots (three per species) on the Mediterranean coast from the southeast of the Iberian Peninsula (Table 1), in areas where the subtropical Mediterranean desert climate is characterised by extreme aridity, with annual rainfall averaging below 250 mm. This lack of rainfall is accompanied by sunshine, with more than 3,000 h per year, and high evapotranspiration^36,37^.

#### Sampling protocol

For sample collection, we collected soil samples and plant leaves or branches in the monospecific plots of all halophytes during the autumn period, which took place between 8 and 15 November 2024, coinciding with the fructification stage. In each plot, we randomly selected three healthy and completely developed adult plants from the center of the species patch.

For each species, three sampling points were selected, and three rhizosediment samples (soil-root interface) were collected from the upper 10 cm of soil at each point by gently shaking the roots. This resulted in a total of 54 samples (9 per species). Every set of three-point samples collected under the same conditions was pooled in a 100 mL plastic bottle, preserved at 4 °C and transported without delay to the lab. Then, the samples were frozen at -80 °C until they were analysed.

For soil nutrient concentration determination, soil samples were air-dried at 37 °C in a forced-air oven for 48 h and subsequently sieved twice through a 2 mm mesh to homogenize and remove debris.

Furthermore, the aerial parts of the three plants (each sample containing three fragments of plant material of 2–3 cm length) were collected and washed with distilled water. Subsequently, samples were dried in a hot air oven at 80 °C for 48 h and homogenized.

### Sample analysis

#### Soil physicochemical measurements.

The pH (Crison pH/mV p-506, Spain) and EC (Crison-522, Spain) were determined in aqueous extracts (1/2.5 and 1/5 w/V, respectively)^38^. Soil texture, including the proportions of sand, silt and clay, was analyzed using the densimeter technique^39^. Oxidizable organic C was determined according to Walkley & Black^40^, and phosphate in solution (Thermo Nicolet Evolution 300 UV-Visible Spectrophotometer, Germany) according to Murphy & Riley^41^. Available P was measured according to Olsen et al.^42^, and determination of total C, N and S was done using the Dumas method (Leco CNS-Trumac, Spain)^43^. In all cases, three replicates were measured for each sample.

#### Plant nutrient concentrations

Tissues from the plants were ground, and 0.5 g subsamples were collected from the aerial parts of three individual plants to create a composite sample for each species plot. Subsamples were digested with 6 mL of HNO_3_, 0.5 mL of HF and 1 mL of H_2_O_2_. Concentrations of the following ions were measured: B^3+^, Ca^2+^, Co^2+^, Cu^2+^, K^+^, Mg^2+^, Mn^2+^, Na^+^, Ni^2+^, Si^4+^ and Zn^2+^; while total elemental concentrations were determined for Fe, Mo, P, S and Se (*n* = 9 per species, 3 replicates per species plot) by inductively coupled plasma (ICP) spectroscopy (ARL-Fison 3410, USA). Total N and C concentrations were determined for undigested dry samples with an elemental analyser (Leco CHNS-932, Spain).

#### Soil phospholipid fatty acids measurements

Phospholipid Fatty Acids (PLFA) analysis was developed to characterize soil microbial communities (*n* = 3 per plot). Analyses were performed on soil samples stored at -80 °C until processing. A gas chromatograph (Agilent 6890 N, USA) equipped with a flame ionization detector (FID) was used for the quantitative and qualitative percentage determination of PLFA. A 100 m long HP-88 capillary column, featuring an internal diameter of 0.25 mm and a stationary phase thickness of 0.2 μm, was employed. H_2_ served as the carrier gas with a flow rate of 2 mL min^− 1^. The oven temperature started at 100 °C and was ramped up by 3 °C per minute until it reached 158 °C. Afterward, the temperature was raised at a rate of 1.5 °C per minute until reaching 190 °C, where it was held constant for 15 min. Then, the temperature increased by 2 °C per minute up to 200 °C. Finally, a faster ramp of 10 °C per minute was applied until reaching 240 °C, which was maintained for 10 min. The injector temperature was set at 300 °C and the detector temperature at 320 °C. Injection was performed using split mode. Fatty acids were identified by comparing their retention times with those in a mixture of Supelco 37 CRM47885 LRAD1-445 standards. PLFA profiles were used to estimate total microbial biomass and the biomass of major microbial groups, including bacteria, fungi and microfauna^44,45^.

#### Soil enzyme activities measurements

Dehydrogenase, arylsulfatase, acid phosphatase, alkaline phosphatase, β-glucosidase and urease activities of the soil samples were determined using a Thermo Scientific Multiskan SkyHigh Microplate Reader (USA) based on the methodology outlined by Dick et al.^46^, Kandeler et al.^47^ and Mulvaney^48^. Specifically, dehydrogenase activity was measured by taking 0.2 g of CaCO_3_ and 20 g of moist soil, that were mixed. From this mixture, 4 g were taken and 1 mL of 3% (w/v) 2,3,5-triphenyl tetrazolium chloride (TTC) solution and 2.5 mL of distilled water were added, and the mixture was incubated for 24 h at 37 °C protected from light. Then, the triphenyl formazan (TPF) formed as a product of the dehydrogenase activity (which is insoluble in water) was extracted with 50 mL of absolute ethanol and its concentration was colorimetrically determined at 485 nm.

For the measurement of arylsulfatase activity, 1 g of moist soil was incubated at 37 °C for 30 min with 4 mL of 0.5 M sodium acetate buffer (pH 5.8) and 1 mL of 50 mM potassium 4-nitrophenyl sulfate. The reaction was halted by the addition of 4 mL of 0.5 M NaOH and 1 mL of 0.5 M CaCl_2_. The amount of 4-nitrophenol produced in the sample assays was measured colorimetrically at a wavelength of 410 nm.

Acid and alkaline phosphatase activities were measured by incubating 1 g of moist soil with 4 mL of MUB buffer (pH 6.3 for acid phosphatases or pH 11 for alkaline phosphatases) and 1 mL of 15 mM disodium 4-nitrophenylphosphate at 37 °C for 30 min. The reaction was stopped with 4 mL of 0.5 M NaOH, and 1 mL of 0.5 M CaCl_2_. The concentration of 4-nitrophenol formed in the sample tests was determined colorimetrically at 410 nm.

β-glucosidase activity was assessed by incubating 1 g of moist soil with 4 mL of MUB buffer at pH 6 and 1 mL of 50 mM 4-nitrophenyl-β-glucopyranoside (β-PNG) for 30 min at 37 °C. The reaction was terminated by adding 4 mL of 0.1 M Tris-H_2_SO_4_ solution and 1 mL of 0.5 M CaCl_2_. The level of 4-nitrophenol produced in the samples was quantified colorimetrically at 410 nm.

To measure urease activity, a soil sample of 1 g was mixed with 4.5 mL of sodium tetraborate buffer adjusted to pH 9.5, and 0.5 mL of 80 mM urea solution at 37 °C for 2 h. The reaction was stopped by adding 6 mL of 1 M KCl solution, which also extracts the ammonium formed in the urease reaction. This was determined colorimetrically at 667 nm according to Mulvaney method^48^. Inactive controls (without the addition of urea in the assays) were used to subtract the ammonium content of the soils.

The moisture content of the soil samples was determined by drying at 105 °C to constant weight, allowing the expression of enzyme activities based on the dry weight of the soil.

For activities involving 4-nitrophenol as a reaction product, in parallel with the active assays, tests were performed on each soil without activity (without adding the reaction substrate to the assays) to remove the soil color.

### Statistical analysis

All multivariate analyses were conducted with R software (v. 4.3.1). Nonmetric Multidimensional Scaling (NMDS; Euclidean distances) was used to describe the overall dissimilarity structure of plant nutrient concentrations, soil properties, and microbial and enzymatic activities, with one outlier removed. Differences among species and plant functional types (PFTs) were tested using PERMANOVA (9999 permutations) and homogeneity of multivariate dispersions was assessed. Homogeneity of multivariate dispersions was specifically tested using PERMDISP to assess whether group differences reflected shifts in centroid location rather than differences in within-group variability. Principal Coordinates Analysis (PCoA) was applied as a complementary, variance-based ordination to visualize species separation and evaluate dispersion patterns, using Bray-Curtis dissimilarities calculated from transformed data. To identify variable-level patterns underlying species grouping, species means of environmental and biochemical variables were transformed to z-scores, Euclidean distances were calculated and hierarchical clustering was performed using complete linkage (k = 3). Clusters differences were tested with PERMANOVA and standardized values were visualized using heatmaps. Potential functional relationships among variables were explored using Spearman’s rank correlations, retaining coefficients with *p* < 0.05 and |r| ≥ 0.6, which were visualized in a customized correlation matrix. Finally, Redundancy Analyses (RDA) were conducted to examine relationships between species, soil physicochemical parameters, enzyme activities and plant nutrients. Model fit was evaluated using both the proportion of variance explained (R^2^) and the adjusted R^2^ to account for the number of explanatory variables. The significance of RDA models, axes and explanatory variables was assessed using permutation tests. Differences among species along RDA axes were evaluated with one-way ANOVA followed by Tukey HSD tests.

## Results

### Species-level differences in leaf or branch nutrient concentrations

**Fig. 1:**
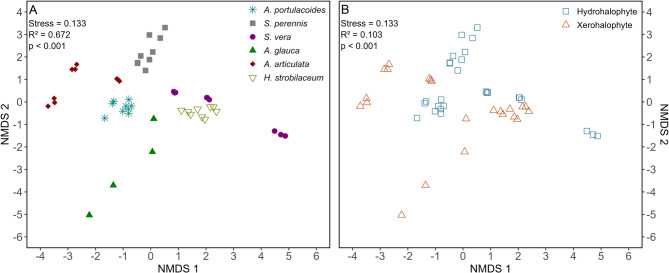
Non-metric multidimensional scaling (NMDS) of standardized plant nutrient concentrations (Euclidean distances). (**A**) Samples colored by species, and (**B**) samples colored by plant functional type (PFTs). Statistical differences among species and PFTs were assessed using PERMANOVA. Corresponding values for stress, R^2^ and p-value are indicated in the corners of each panel.

The NMDS ordination of standardized nutrient concentrations data revealed clear clustering of species in multivariate space (Fig. 1), with a low stress value (stress = 0.133), indicating that the reduced-dimensional plot provides a reasonable representation of pairwise dissimilarities among samples. PERMANOVA analyses, conducted on the same distance matrix used for NMDS ordination, showed that nutrient concentrations differed significantly among species (Fig. 1A; *p* < 0.001, R^2^ = 0.672) and between plant functional types (PFTs) (Fig. 1B; *p* < 0.001, R^2^ = 0.103). While the difference between PFTs is statistically significant, the low R^2^ indicates that PFT explains only a small portion of the total variation, whereas species identity explained a substantially larger fraction of the variance. Tests of multivariate dispersion (PERMDISP) indicated no significant heterogeneity among PFTs (Fig. 1B; *p* > 0.05, F = 1.45), suggesting that the observed differences reflect genuine shifts in group centroids rather than differences in within-group variability. In contrast, some heterogeneity in dispersion was detected among species (Fig. 1A; *p* < 0.001, F = 6.93); however, the high proportion of variation explained by species identity suggests that observed differences among species likely reflect distinct positions of species centroids in multivariate space, rather than dispersion alone. Given that each species was sampled at distinct geographic locations (Table 1), species effects cannot be completely disentangled from site-specific environmental conditions.

### Multivariate differentiation among species

**Fig. 2:**
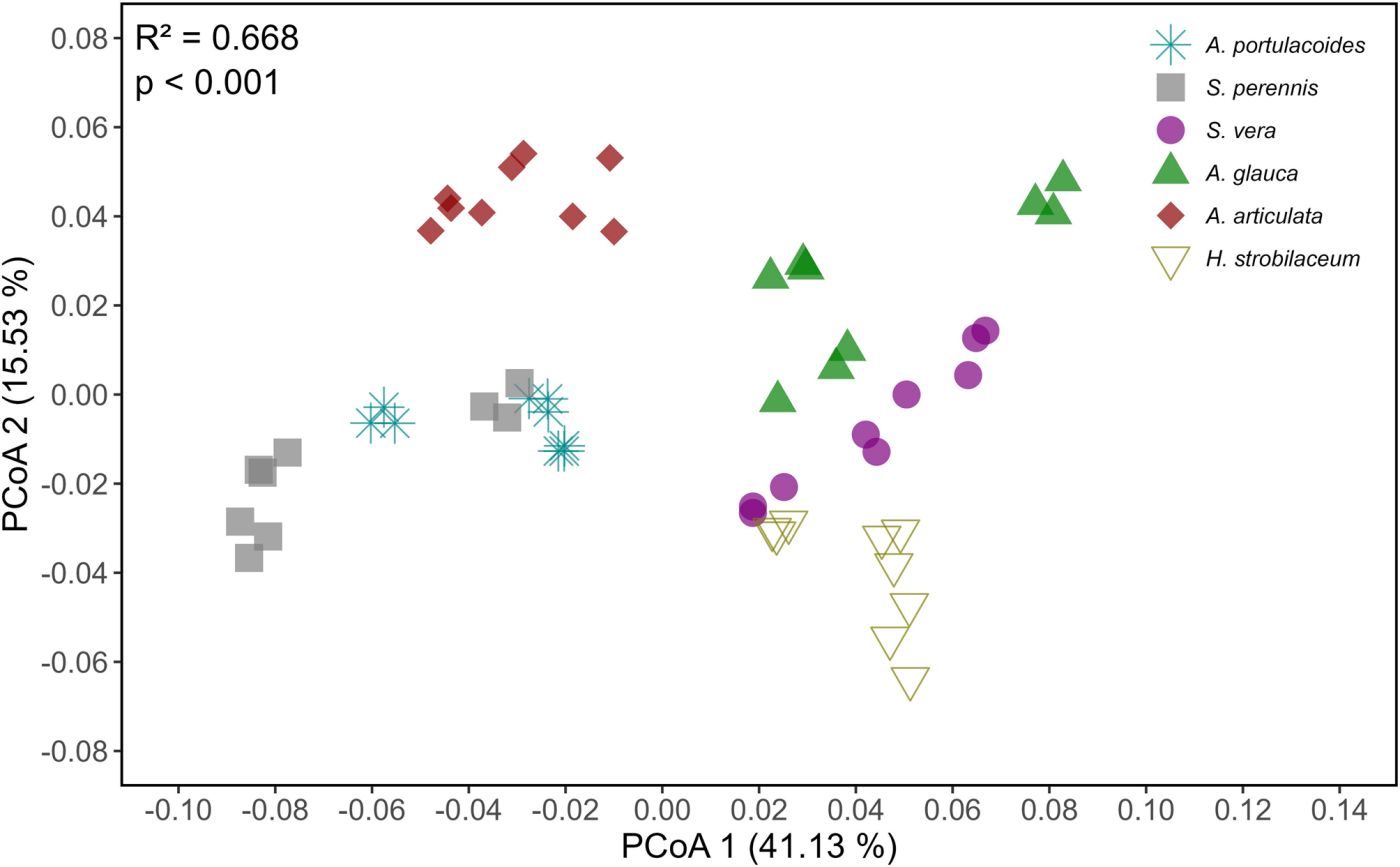
Principal Coordinates Analysis (PCoA) of standardized plant and soil variables, including nutrient concentrations, soil physicochemical properties, soil microbial composition and enzymatic activities. Points represent individual samples, colored by species. Statistical differences among species were assessed using PERMANOVA (Bray-Curtis). Corresponding values for R^2^ and p-value are indicated in the figure corner.

The PERMANOVA based on Bray-Curtis dissimilarity revealed a significant effect of species (Fig. 2; *p* < 0.001, R^2^ = 0.668), indicating that 66.8% of the multivariate variation was explained by species identity. The test for homogeneity of group dispersions (i.e., multivariate spread) was not significant, confirming that the differences detected by PERMANOVA were not driven by unequal within-group dispersion. The PCoA ordination showed clear separation among species, although some overlap was observed between *A. portulacoides* and *S. perennis*, as well as between *A. glauca*, *H. strobilaceum* and *S. vera*. In contrast, *A. articulata* was separated from the other two groups in multivariate space (Fig. 2).

### Multivariate grouping of species based on environmental and biological variables

**Fig. 3:**
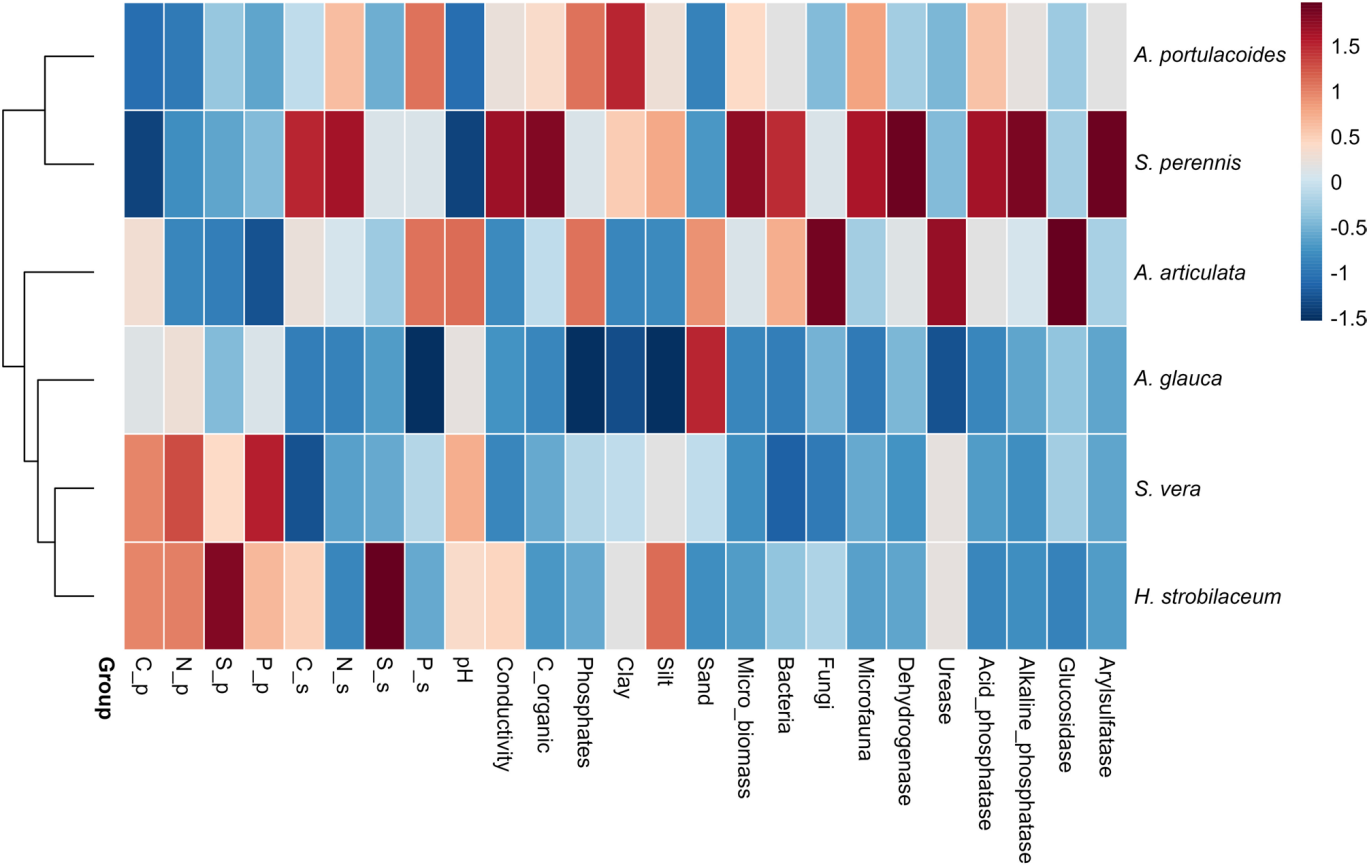
Heatmap of standardized plant and soil variables, including nutrient concentrations, soil physicochemical properties, soil microbial composition and enzymatic activities. Species were grouped into three clusters based on hierarchical clustering using Euclidean distances (complete linkage). Colors represent standardized values (blue = low, red = high). Variables ending in “_p” refer to plant nutrient concentrations, whereas “_s” denotes soil nutrient concentrations. “C_organic” refers to soil organic carbon, and “Micro_biomass” indicates microbial biomass. PERMANOVA confirmed significant differences among clusters (*p* < 0.05, R^2^ = 0.695).

Hierarchical clustering based on standardized environmental and biological variables grouped the six species into three distinct clusters (Fig. 3). The heatmap revealed differences among species in nutrient accumulation, microbial and enzymatic activity, and soil physicochemical conditions. Species within Group 1 (*A. portulacoides* and *S. perennis*) showed higher values for microbial biomass and enzymatic activity; while Group 2 (*A. articulata*) was characterized by intermediate to low values across most variables. Group 3 (*A. glauca*, *H. strobilaceum* and *S. vera*) tended to have lower levels of microbial and enzymatic indicators, but with higher plant C-N-S-P concentrations. This differentiation was statistically supported by PERMANOVA results (Fig. 3; *p* < 0.05, R^2^ = 0.695), indicating significant multivariate differences in environmental variables between groups.

### Correlation analysis of environmental and biological variables

**Fig. 4:**
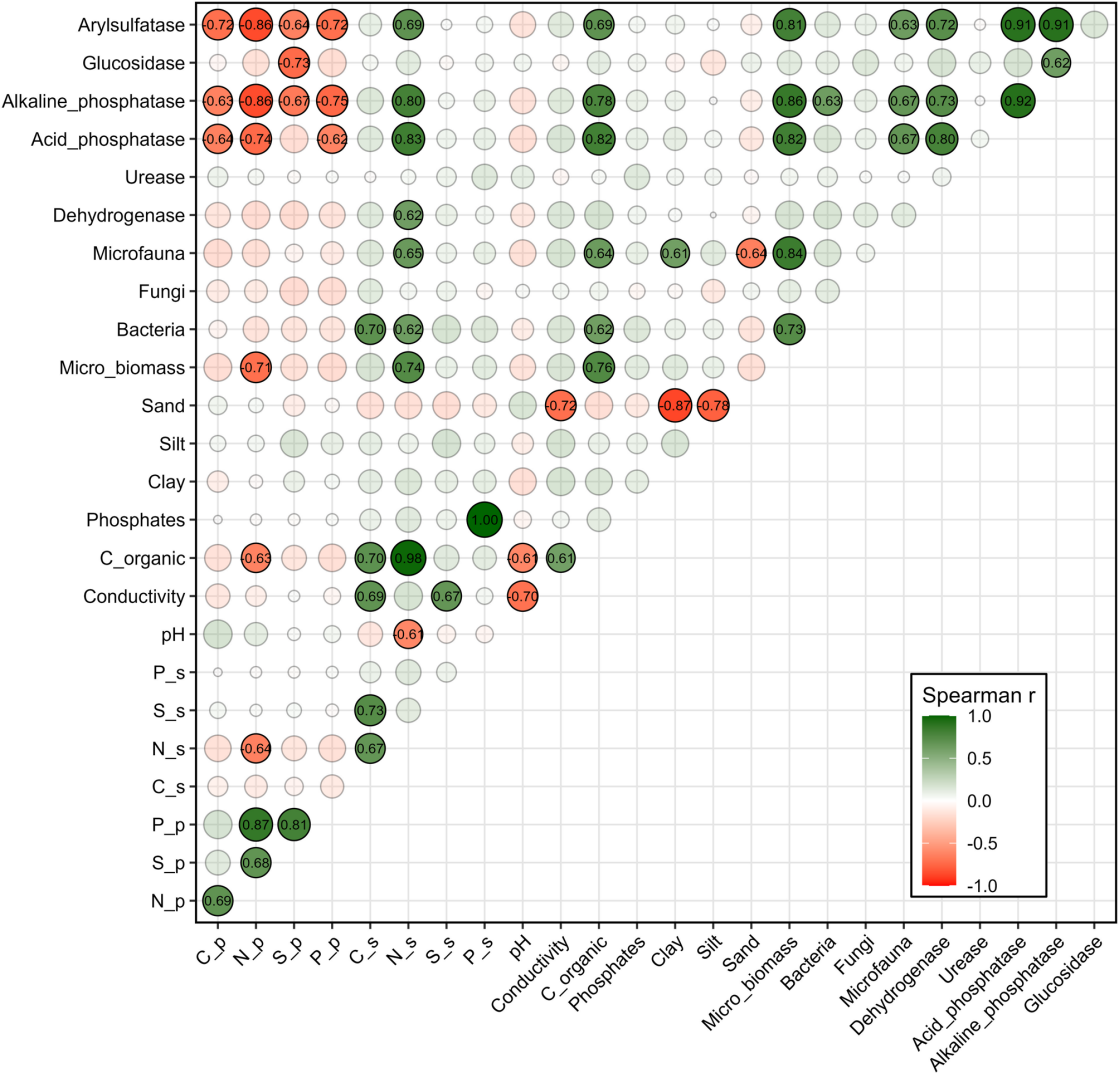
Spearman correlation matrix including standardized plant and soil variables, as nutrient concentrations, soil physicochemical properties, soil microbial composition and enzymatic activities. Point size and color indicate the strength and direction of the correlation (green: positive, red: negative). Transparent circles correspond to non-significant correlations. Variables ending in “_p” refer to plant nutrient concentrations, whereas “_s” denotes soil nutrient concentrations. “C_organic” refers to soil organic carbon, and “Micro_biomass” indicates microbial biomass. Significant and strong correlations (*p* < 0.05 and |r| ≥ 0.6) are labeled with their respective coefficient values.

The correlation matrix (Fig. 4) revealed distinct associations among soil, plant, microbial and enzymatic variables. Relationships among variables were assessed using Spearman’s rank correlation, considering only coefficients that were both statistically significant (*p* < 0.05) and strong (|r| ≥ 0.6). Strong positive correlations were observed, for instance, between soil organic C and N in soil with enzymatic activities and microbial biomass. Additionally, a positive correlation was observed between microbial biomass, particularly microfauna, and enzymatic activities such as arylsulfatase and acid and alkaline phosphatases. Conversely, plant nutrients (i.e. C, N, S and P) showed a negative correlation with specific enzymatic indicators, such as soil arylsulfatase and alkaline phosphatase activities. The use of a Spearman correlation allowed detection of both linear and monotonic relationships among diverse variables.

### Influence of soil physicochemical properties on enzymatic activity and microorganisms

**Fig. 5:**
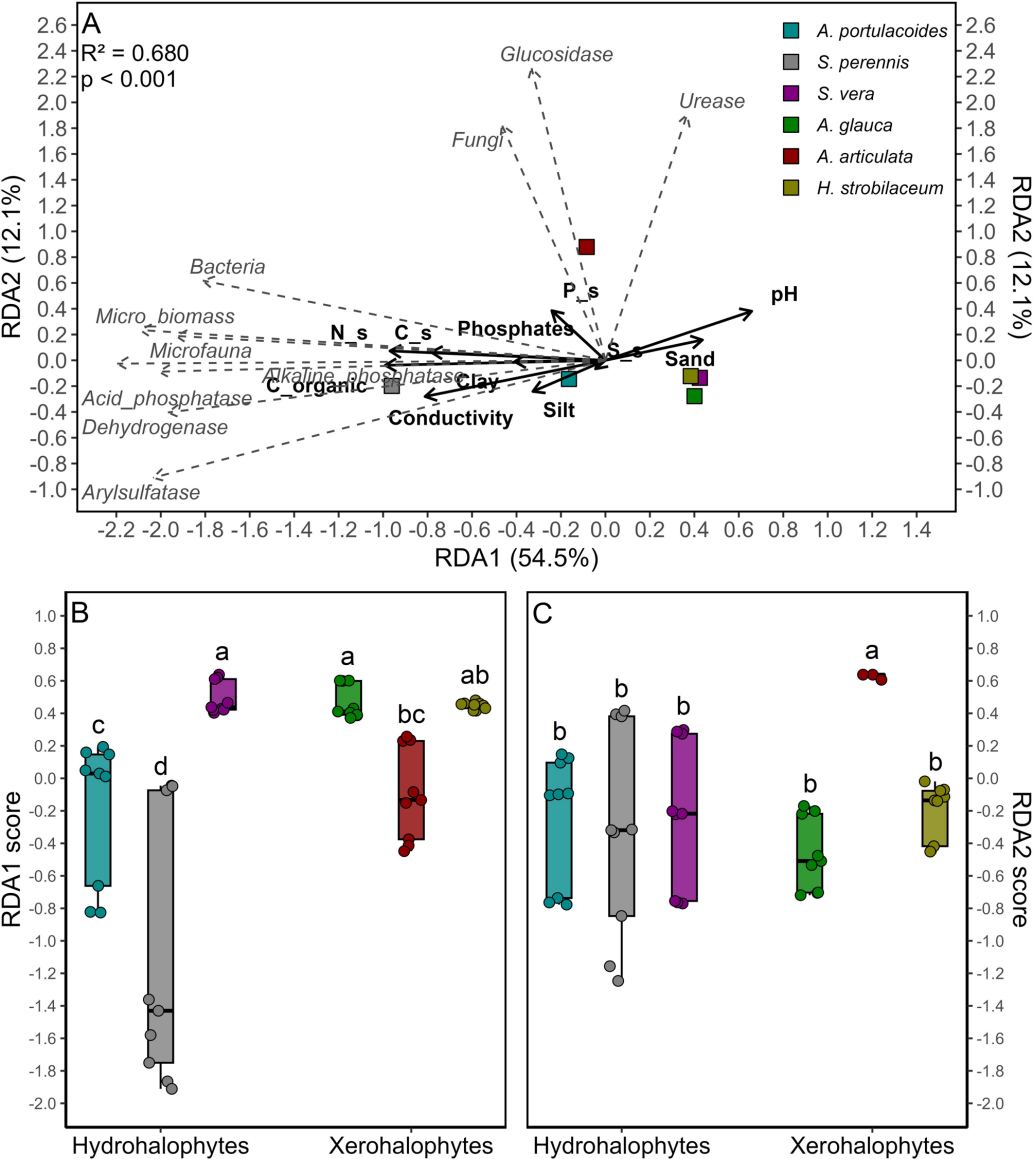
(**A**) Redundancy analysis (RDA) showing the relationships between soil enzymatic activities and microbial composition (dashed grey arrows) and soil physicochemical properties (solid black arrows). Squares represent centroid species, colored by species. Axes indicate the variation in soil enzymatic activities and microbial composition explained by the soil physicochemical properties. The adjusted coefficient of determination (R^2^) and the permutation-based p-value of the global RDA model are shown in the panel. (**B**) Distribution of RDA1 and (**C**) RDA2 scores for each plant species, grouped into hydrohalophytes and xerohalophytes. Scores represent variation in enzymatic activities and soil microbial composition constrained by soil physicochemical properties. Boxplots show median and interquartile range; jittered points indicate individual samples. Variables ending in “_s” denotes soil nutrient concentrations. “C_organic” refers to soil organic carbon, and “Micro_biomass” indicates microbial biomass. Letters above boxes indicate statistically significant differences among species based on Tukey’s HSD post-hoc test (*p* < 0.05).

Redundancy Analyses (RDA) and permutation tests indicated that soil physicochemical properties explained 66.6% of the variation in microbial composition and enzymatic activities (Fig. 5A; *p* < 0.001, R^2^ = 0.680). Analysis of individual explanatory variables showed that soil pH and conductivity and contents of organic C, phosphates, clay, silt and S significantly influenced soil biological activity (*p* < 0.05), whereas sand content and soil C-N-P did not have a statistically significant effect.

Biplots revealed a clear segregation of species according to microbial composition and enzymatic activities. Statistical differences among species were assessed using one-way ANOVA followed by Tukey HSD tests. Along RDA1, significant differences were observed among species (Fig. 5B; *p* < 0.001), with those positioned at the most negative end, such as *S. perennis* and *A. portulacoides*, being associated with higher microbial biomass and enzymatic activity levels. In contrast, along RDA2, only *A. articulata* differed significantly (Fig. 5C; *p* < 0.001), showing a strong association with increased urease and glucosidase activities and fungal abundance.

### Influence of soil physicochemical properties on the plant nutritional status

**Fig. 6:**
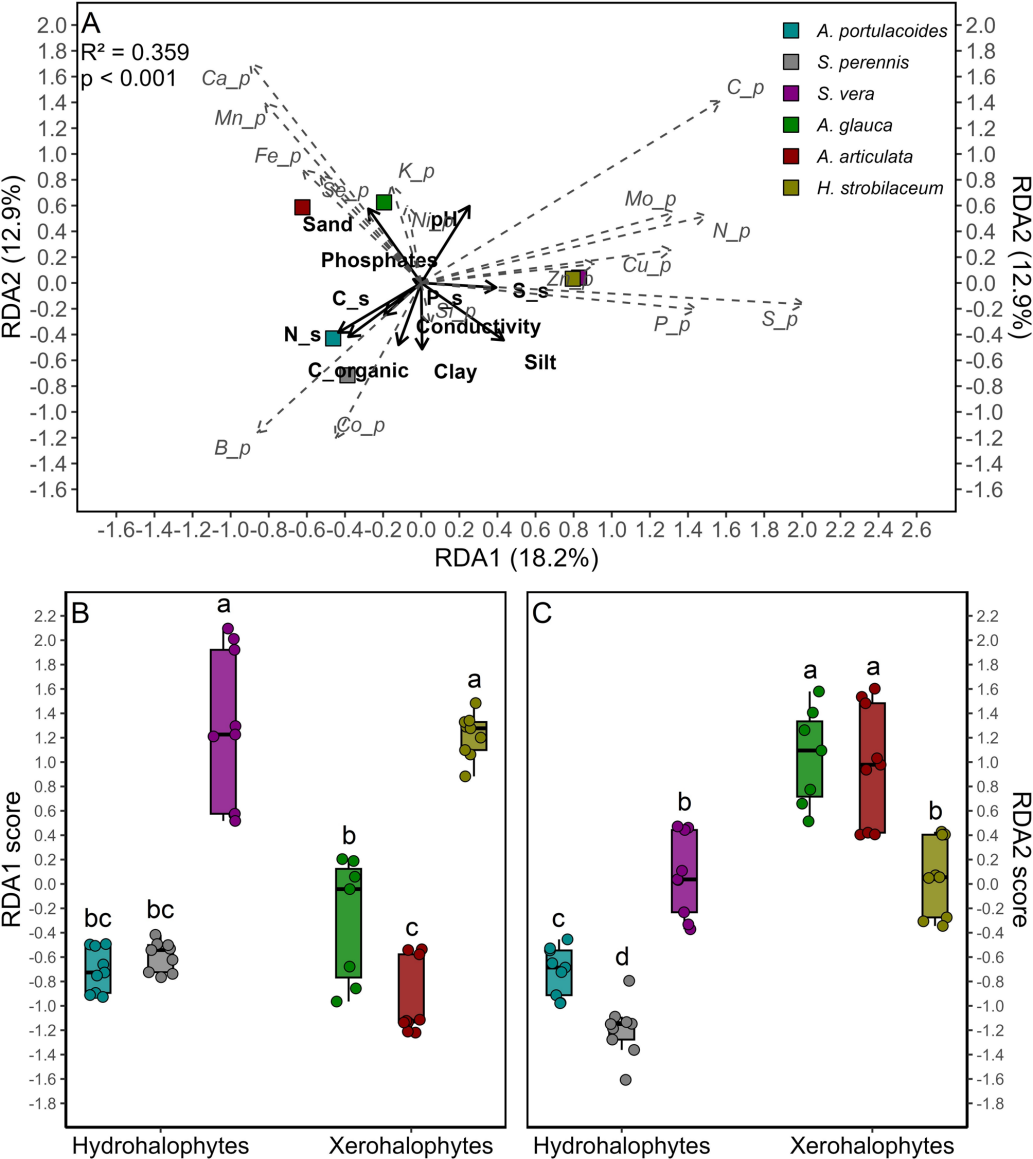
(**A**) Redundancy analysis (RDA) showing the relationships between plant nutrient concentrations (dashed grey arrows) and soil physicochemical properties (solid black arrows). Squares represent centroid species, colored by species. Axes represent the variation in plant nutrient concentrations explained by soil physicochemical properties. The adjusted coefficient of determination (R^2^) and the permutation-based p-value of the global RDA model are shown in the panel. (**B**) Distribution of RDA1 and (**C**) RDA2 scores for each plant species, grouped into hydrohalophytes and xerohalophytes. Scores represent variation in plant nutrient composition constrained by soil physicochemical properties. Boxplots show median and interquartile range; jittered points indicate individual samples. Variables ending in “_p” refer to plant nutrient concentrations, whereas “_s” denotes soil nutrient concentrations. “C_organic” refers to soil organic carbon. Letters above boxes indicate statistically significant differences among species based on Tukey’s HSD post-hoc test (*p* < 0.05).

RDA and permutation tests showed that soil physicochemical properties accounted for 31.1% of the variation in plant nutrient concentrations (Fig. 6A; *p* < 0.001, R^2^ = 0.359). Analysis of individual explanatory variables indicated that soil pH and conductivity, and organic C, silt and clay contents had a significant effect on plant nutrient concentrations (*p* < 0.05), whereas phosphates, sand content, and soil C-N-P-S did not show a statistically significant influence.

Biplot analysis highlighted a noticeable separation among plant species according to their nutrient concentrations. Statistical differences among species were assessed using one-way ANOVA followed by Tukey HSD tests. Along the RDA1 axis, species differed significantly (Fig. 6B; *p* < 0.001), with *S. vera* and *H. strobilaceum* associated with higher plant C-N-S-P concentrations, appearing at the positive extreme. In contrast, the other four species were positioned toward the negative side, associated with higher concentrations of Ca^2+^, Mg^2+^ and Mn^2+^. Along RDA2, *A. glauca* and *A. articulata* exhibited higher values at the positive end of the axis, associated with higher concentrations of Ca^2+^, Mn^2+^ and Fe. In contrast, *A. portulacoides* and *S. perennis* were located at the opposite end, associated with higher B^3+^ and Co^2+^ content. And *S. vera* and *H. strobilaceum* occupied an intermediate position (Fig. 6C; *p* < 0.001).

### Influence of soil enzymatic activity and microorganisms on the plant nutritional status

**Fig. 7:**
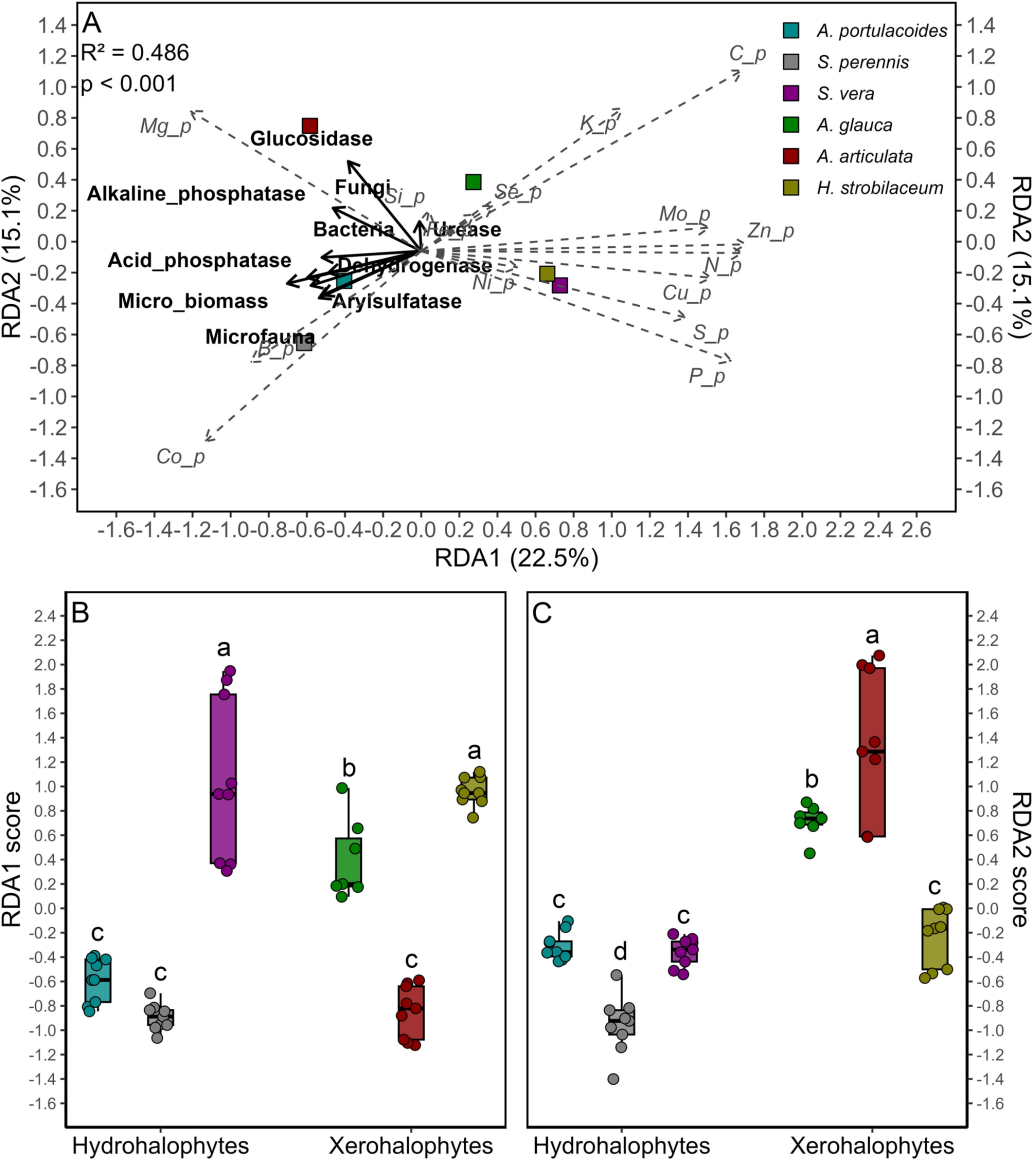
(**A**) Redundancy analysis (RDA) showing the relationships between plant nutrient concentrations (dashed grey arrows) and soil enzymatic activities and microbial composition (solid black arrows). Squares represent centroid species, colored by species. Axes represent the variation in plant nutrient concentrations explained by soil enzymatic activities and microbial composition. The adjusted coefficient of determination (R^2^) and the permutation-based p-value of the global RDA model are shown in the panel. (**B**) Distribution of RDA1 and (**C**) RDA2 scores for each plant species, grouped into hydrohalophytes and xerohalophytes. Scores represent variation in plant nutrient composition constrained by soil enzymatic activities and microbial composition. Boxplots show median and interquartile range; jittered points indicate individual samples. Variables ending in “_p” refer to plant nutrient concentrations, and “Micro_biomass” indicates microbial biomass. Letters above boxes indicate statistically significant differences among species based on Tukey’s HSD post-hoc test (*p* < 0.05).

RDA and permutation tests indicated that soil microbial and enzymatic variables explained 37.6% of the variation in plant nutrient concentrations (Fig. 7A; *p* < 0.001, R^2^ = 0.486). Analysis of individual explanatory variables showed that dehydrogenase, urease and acid phosphatase activities, together with bacterial and fungal abundances, had significant effects on plant nutrient concentrations (*p* < 0.05).

Biplots revealed a clear segregation of species according to their nutrient profiles. Statistical differences among species were assessed using one-way ANOVA followed by Tukey HSD tests. Along RDA1, significant differences were observed among species (Fig. 7B; *p* < 0.001), with *S. vera* and *H. strobilaceum* located at the positive end of the axis, associated with higher plant nutrient concentrations; while *A. portulacoides*, *S. perennis* and *A. articulata* were located at the opposite end, linked to lower nutrient concentrations. Along RDA2, *A. articulata* and *A. glauca* were clearly differentiated, reflecting associations with higher C, K^+^ and Mg^2+^ concentrations, whereas the remaining species occupied intermediate positions (Fig. 7C; *p* < 0.001).

## Discussion

Plant functional types (PFTs) classify species according to their structural, physiological and/or phenological characteristics, reflecting both their responses to environmental conditions and their functional roles within ecosystems^49,50^. These classifications thus help simplify ecological complexity and may improve our understanding of plant responses to environmental change and human disturbance^51^. The influence of both abiotic and biotic factors on the distribution of different PFTs is well documented, with several studies reporting large variations associated with environmental conditions^52,53^ and biological interactions such as soil microbiota or enzyme activity^54^. Moreover, plant functional traits and soil microbiota interactions are essential for nutrient cycling and ecosystem functioning, which are thought to influence plant nutrition^55,56^. It is well established that soil physicochemical properties not only influence plant nutritional status directly but also are associated with variation in soil enzymatic activity and microbial communities, which in turn influence plant nutrient acquisition^57,58^. On the other hand, each species is adapted to soils with specific physicochemical conditions that enable nutrient availability and uptake by plants^59^; and nutrient concentrations could be influenced by the interplay between genetic predisposition and environmental factors, such as soil composition and salinity^60^. This is supported by studies showing that halophytes have developed distinct mechanisms to manage salinity stress, including ion transport mechanisms and compatible solute accumulation, and are associated with diverse nutrient profiles^61–63^. Therefore, this study aims to elucidate whether this complex network of interactions produces a consistent nutritional response within species belonging to the same PFT or whether these patterns are species-specific.

Overall, our results indicated that halophyte nutritional status was more closely related to variation among species than to PFT classification as hydro- or xero-halophytes, as PFTs explain only a small fraction of the overall variability (Fig. 1). Although PFT differences were statistically significant, their explanatory power was limited compared to species identity (Fig. 1B). However, it is important to acknowledge that each species was sampled from distinct geographic locations and species did not co-occur within the same sites (Table 1). Therefore, observed species-level patterns likely reflect combined species-site effects rather than intrinsic species traits alone (Fig. 2).

The halophytes *A. portulacoides* and *S. perennis* displayed highly similar nutritional status, consistent with their classification as hydrohalophytes (Fig. 1A), despite the different local soil physicochemical conditions. For detailed information on each measured variable, please refer to the Supplementary Material. Several studies have highlighted the importance of soil physicochemical properties in nutrient acquisition^59,64^. Soil physicochemical are associated with variation in plant biochemical pathways, and are linked to diverse nutrient profiles^65,66^. In this way, soil texture influences a wide range of other soil characteristics: water retention, permeability specific surface area^67^, organic matter stability, aeration, compaction, conductivity and pH^68^. In turn, these properties regulate microbial diversity and enzyme performance within coastal saline soil environments, and consequently nutrient cycling^69^. Thus, the soil microbiome may contribute to soil fertility and nutrient cycling^58,70^. The microorganisms decompose organic matter, recycle nutrients and facilitate mineral weathering, providing essential elements and building blocks for plant nutrition and the synthesis of vital compounds in all living organisms^71,72^.

In our study, pH, conductivity, and soils clay, silt and organic C contents were the most determining environmental factors in explaining the nutritional status of the plants; and silt and clay textures were associated with higher soil microbial biomass and enzymatic activity compared with sandy soils. Besides, microorganisms and enzymatic activities were more abundant in neutral pH soils with high conductivity, organic C and phosphate contents (Fig. 6). These findings align with Chaudhary et al.^63^, who reported that rhizosphere soils in salt marshes with high organic C, nutrient availability and salinity exhibited greater bacterial diversity and enzyme activity. According to our results, there was a positive correlation between soil nutrient content and microbial abundance (Fig. 5), highlighting the interplay between microbial biomass, enzymatic activity and soil nutrients such as N and organic C; which has also been documented by other authors^73,74^. In contrast, we also observed a negative correlation between enzymatic activity and plant nutrient content (Fig. 4). These correlations do not imply causality and may reflect indirect effects or shared environmental drivers. The hydrohalophytes *S. perennis* and *A. portulacoides* exhibited high enzymatic activity and microbial abundance (especially microfauna) in their soils but low nutrient concentrations in their tissues (Figs. 3 and 7). In this way, the heatmap analysis showed that these two species form a distinct cluster characterized by these variables, indicating their key role in driving functional similarity (Fig. 3). Studies indicate that higher enzymatic activity is often associated with lower nutrient concentrations in plant tissues, which may reflect that enzymes may be more active in nutrient-poor conditions, facilitating nutrient mobilization from organic matter^75^. Otherwise, other studies support that enzymatic activities differ widely among wetland species and were weakly related to nutrient concentrations^76^.

On the other hand, the xerohalophytes *A. glauca* and *H. strobilaceum*, and the hydrohalophyte *S. vera* were characterized by higher tissue C, N, P and S concentrations, but lower levels of soil enzymatic activities and microbial biomass (Figs. 3 and 7). Their soils were characterized by higher pH values ​​and lower organic C and nitrogen contents. Finally, *A. articulata* appeared to form an independent group regarding its nutritional profile, which was conditioned by sandy soils and high enzymatic activities, indicating a unique ecological niche (Fig. 3). Thus, studies of nutrient stoichiometry at the species level can help clarify plant-environment relationships and nutrient cycling patterns in ecosystems^77^.

At present, no specific studies directly compare the nutritional mechanisms among different halophytes. To better understand the differences among the six species included in this study, we highlight some of their most notable physiological and morphological traits. *A. portulacoides*, a woody shrub with slightly fleshy leaves, shows high relative growth rates under moderate salinity and utilizes osmolytes such as betaine and proline for osmotic adjustment, which likely influence its tissue nutrient profiles and salt compartmentalization^78^. Its preference for oxygenated soils aligns with the absence of root aerenchyma and its ability to sequester metals in cell walls, contributing to nutrient regulation^79^. *S. perennis* forms dense mats with succulent stems and a robust root system adapted to resist sediment penetration and hydraulic stress^80^. Both species share a preference for relatively well-drained coastal salt marsh sediments and often co-occur in similar salt-marsh plant communities, suggesting overlapping ecological niches and exposure to comparable soil physicochemical conditions. In our study, both species consistently showed lower tissue nutrient concentrations compared to the other halophytes, despite growing in soils with high microbial biomass and enzymatic activity. This result could be consistent with nutrient dilution effects due to rapid biomass accumulation, or with species-level physiological traits affecting nutrient uptake and allocation. In contrast, *S. vera* demonstrates marked anatomical plasticity, including sclerification of vascular tissues and broader metaxylem vessels, that facilitate nutrient transport under high salinity. Its root biomass increases with salinity, enhancing water and nutrient uptake^81^. This hyper-accumulator’s adaptation likely explains its higher tissue concentrations of C, N, P and S compared to other hydrohalophytes. Additionally, *S. vera* grows in alkaline soils typical of desert xerohalophytes, distinguishing it from other species^82^.

*H. strobilaceum* thrives in extreme saline soils by accumulating high mineral content and actively assimilating salts to reduce soil salinity. Specialized water storage tissues surrounding its roots support survival in hypersaline depressions, and its ability to actively absorb and sequester salts may alter local soil salinity and nutrient dynamics^83,84^. Meanwhile, *A. glauca* inhabits arid, poorly structured soils and may possess unique nutrient acquisition strategies, although detailed physiological data are lacking^85^. Finally, *A. articulata*, a slow-growing stem-succulent shrub with C4 metabolism, remains poorly studied in nutrient physiology but is known for rich secondary metabolite production, reflecting specialized adaptations to arid saline environments^86,87^.

Together, these differences among species in morphological, physiological and biochemical traits, in combination with environmental factors, are likely to contribute to variation in nutrient stoichiometry and cycling. This supports the view that species identity is an important factor relative to broad functional classifications in shaping halophyte nutritional status. Further comparative physiological studies among halophyte species are needed to deepen our understanding of the underlying mechanisms driving their diverse nutrient strategies and adaptations.

## Conclusion

Plant functional type (PFT) traits determine both the changes that plants induce in the soil and their responses to these changes, making them central to understanding and generalizing variation in plant-soil feedback^88^. However, our study demonstrated that halophyte nutrient status was generally related to species identity and local soil conditions, rather than being explained by their classification into hydro- or xerohalophytes. Only the hydrohalophytes *A. portulacoides* and *S. perennis* displayed similar nutritional patterns regardless of differences in local soil physicochemical conditions. Thus, our findings revealed a specific pattern of nutrient acquisition that cannot be fully explained by PFTs. Distinct patterns of nutrient concentrations among species were strongly linked to soil physicochemical properties such as pH, EC, soil texture and organic C content, as well as to microbial abundance and soil enzymatic activities. High salinity and alkaline conditions were associated with lower nutrient concentrations in some species, whereas neutral pH and fine-textured soils were associated with higher microbial biomass and enzymatic activity. Moreover, a negative correlation was observed between soil enzyme activity and the nutrient content in plants. These results underscore the complexity of interactions among plants, soil and microbes in saline habitats. This highlights the need to better understand species-specific traits and their interactions with soil processes for predicting halophyte responses to environmental change and guiding management in coastal saline ecosystems.

## Supplementary Information

Below is the link to the electronic supplementary material.

Supplementary Material 1

## Data Availability

The data collected for this study is available at: https:/doi.org/10.12795/11441/177215 and https:/hdl.handle.net/11441/177215.
