# Supplementary material for: Species-specific traits determine halophyte nutrient patterns rather than plant functional types
Source: Sci Rep. 2026 Apr 21;16:18477. doi: 10.1038/s41598-026-49735-1 (PMC13265776; doi:10.1038/s41598-026-49735-1)
Supplement: Supplementary file 1 — Supplementary Material 1 [file 41598_2026_49735_MOESM1_ESM.docx]

|  | **B^3+^** | **Ca^2+^** | **Co^2+^** | **Cu^2+^** | **Fe** | **K^+^** | **Mg^2+^** | **Mn^2+^** | **Mo** | **Na^+^** | **Ni^2+^** | **Se** | **Si^4+^** | **Zn^2+^** |
| --- | --- | --- | --- | --- | --- | --- | --- | --- | --- | --- | --- | --- | --- | --- |
| **Species** | mg kg^-1^ | % | mg kg^-1^ | mg kg^-1^ | mg kg^-1^ | % | % | mg kg^-1^ | mg kg^-1^ | % | mg kg^-1^ | mg kg^-1^ | mg kg^-1^ | mg kg^-1^ |
| *A. portulacoides* | 48 ±4.6^a^ | 0.7 ±0.03^a^ | 0.2 ±0.01^a^ | 2.0 ±0.09^a^ | 219 ±43^a^ | 1.8 ±0.0^a^ | 0.9 ±0.03^a^ | 75 ±4.6^ab^ | - | 9 ±0.2^a^ | 1.3 ±0.33^a^ | - | 330 ±56^ab^ | 24 ±1.3^a^ |
| *S. perennis* | 20 ±2.4^b^ | 0.4 ±0.02^a^ | 39 ±8.6^b^ | 3.5 ±0.26^a^ | 152 ±37^a^ | 0.9 ±0.04^b^ | 0.7 ±0.02^b^ | 72 ±4.1^ab^ | - | 10 ±0.3^b^ | 2.7 ±0.69^a^ | - | 271 ±66^ab^ | 10 ±0.6^b^ |
| *S. vera* | 16 ±1.6^bc^ | 0.3 ±0.02^a^ | 0.2 ±0.02^a^ | 13 ±1.4^b^ | 132 ±16^a^ | 1.4 ±0.05^c^ | 0.3 ±0.02^c^ | 47 ±2.8^a^ | 0.5 ±0.18^a^ | 7 ±0.4^c^ | 1.8 ±0.23^a^ | - | 189 ±28^a^ | 26 ±3.1^a^ |
| *A. glauca* | 14 ±2.9 ^bc^ | 1.0 ±0.02^a^ | 1.3 ±0.44^a^ | 5.8 ±0.55^c^ | 900 ±265^b^ | 2.2 ±0.16^d^ | 1.0 ±0.07^a^ | 141 ±3.9^bc^ | 3.3 ±2.5^a^ | 6 ±0.1^c^ | 6.9 ±1.79^b^ | 1.7 ±1.29^a^ | 369 ±15^b^ | 30 ±2.0^a^ |
| *A. articulata* | 20 ±0.4^b^ | 3.4 ±0.5^b^ | 0.2 ±0.03^a^ | 2.2 ±0.19^a^ | 151 ±30^a^ | 1.4 ±0.12^c^ | 0.9 ±0.07^a^ | 187 ±43.7^c^ | - | 4 ±0.2^d^ | 0.9 ±0.12^a^ | - | 263 ±14^ab^ | 10 ±1.7^b^ |
| *H. strobilaceum* | 9 ±0.5^c^ | 0.4 ±0.01^a^ | 0.5 ±0.12^a^ | 7.8 ±0.18^c^ | 230 ±12^a^ | 1.5 ±0.05^c^ | 0.3 ±0.02^c^ | 80 ±3.4^ab^ | 0.7 ±0.02^a^ | 8 ±0.3^a^ | 3.5 ±0.49^a^ | - | 420 ±12^b^ | 26 ±0.8^a^ |

**Table S1.** Boron, calcium, cobalt, copper, iron, potassium, magnesium, manganese, molybdenum, sodium, nickel, selenium, silicon, and zinc concentrations in aerial tissues of hydrohalophytes (*Atriplex portulacoides*, *Salicornia perennis*, and *Suaeda vera*) and xerohalophytes (*Atriplex glauca*, *Anabasis articulata*, and *Halocnemum strobilaceum*) species. Each value represents the mean of nine replicates (n = 3 per plot) ± SE. Different lowercase letters indicate means that are significantly different among species within each nutrient concentration, based on ANOVA followed by Tukey’s HSD post-hoc test (p < 0.05).


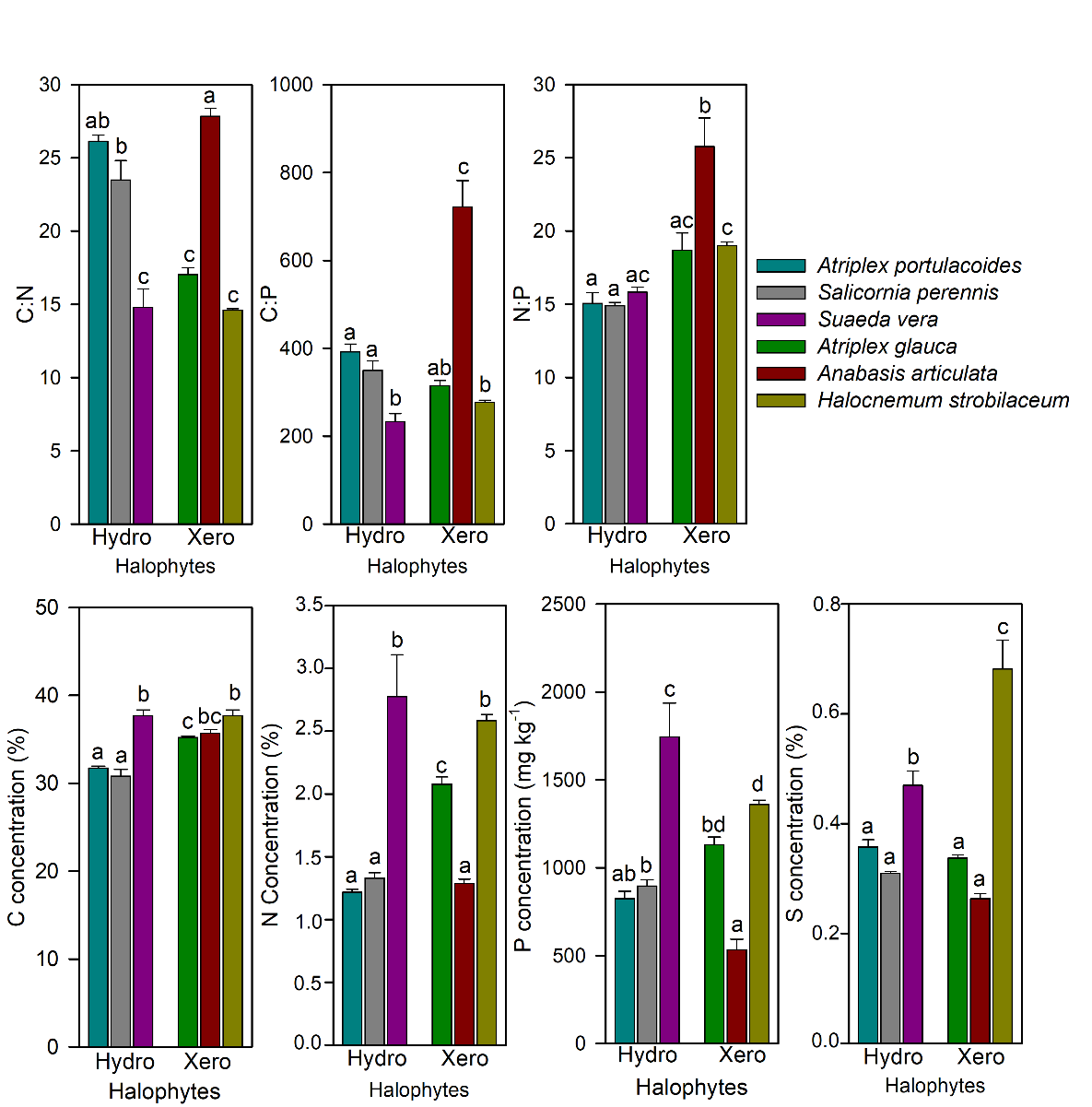


**Figure S1.** C:N, C:P, and N:P ratios, and carbon, nitrogen, phosphorous, and sulfur concentrations in aerial tissues of hydrohalophyte and xerohalophyte species. Each value represents the mean of nine replicates (n = 3 per plot) ± SE. Different lowercase letters indicate means that are significantly different among species, based on ANOVA followed by Tukey’s HSD post-hoc test (p < 0.05).


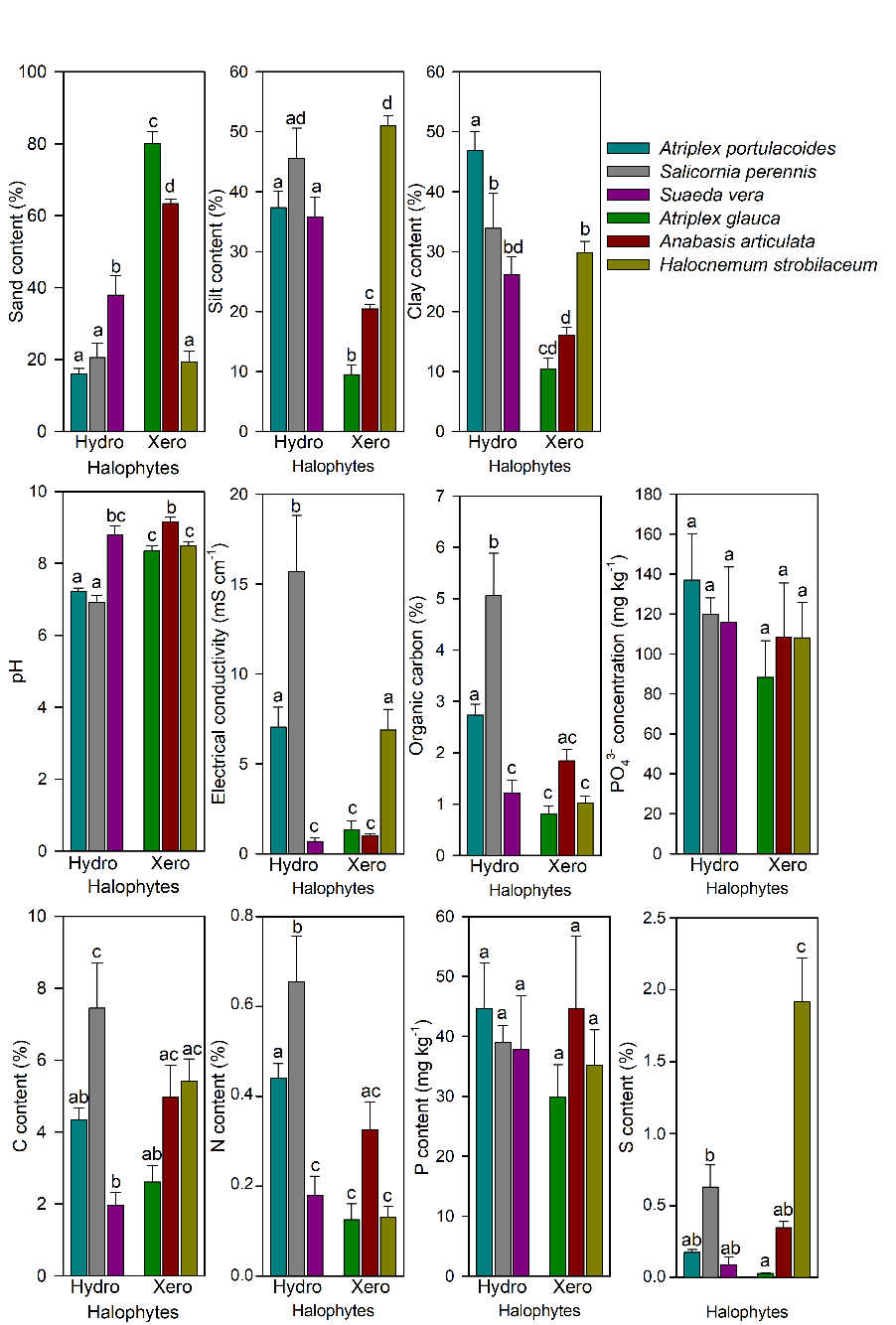
**Figure S2.** Percentages of sand, silt and clay, pH, electrical conductivity, Percentage of organic carbon, and phosphate concentration of sampled soils in the rhizosphere of hydrohalophyte and xerohalophyte species. Each value represents the mean of nine replicates (n = 3 per plot) ± SE. Different lowercase letters indicate means that are significantly different among species, based on ANOVA followed by Tukey’s HSD post-hoc test (p < 0.05).


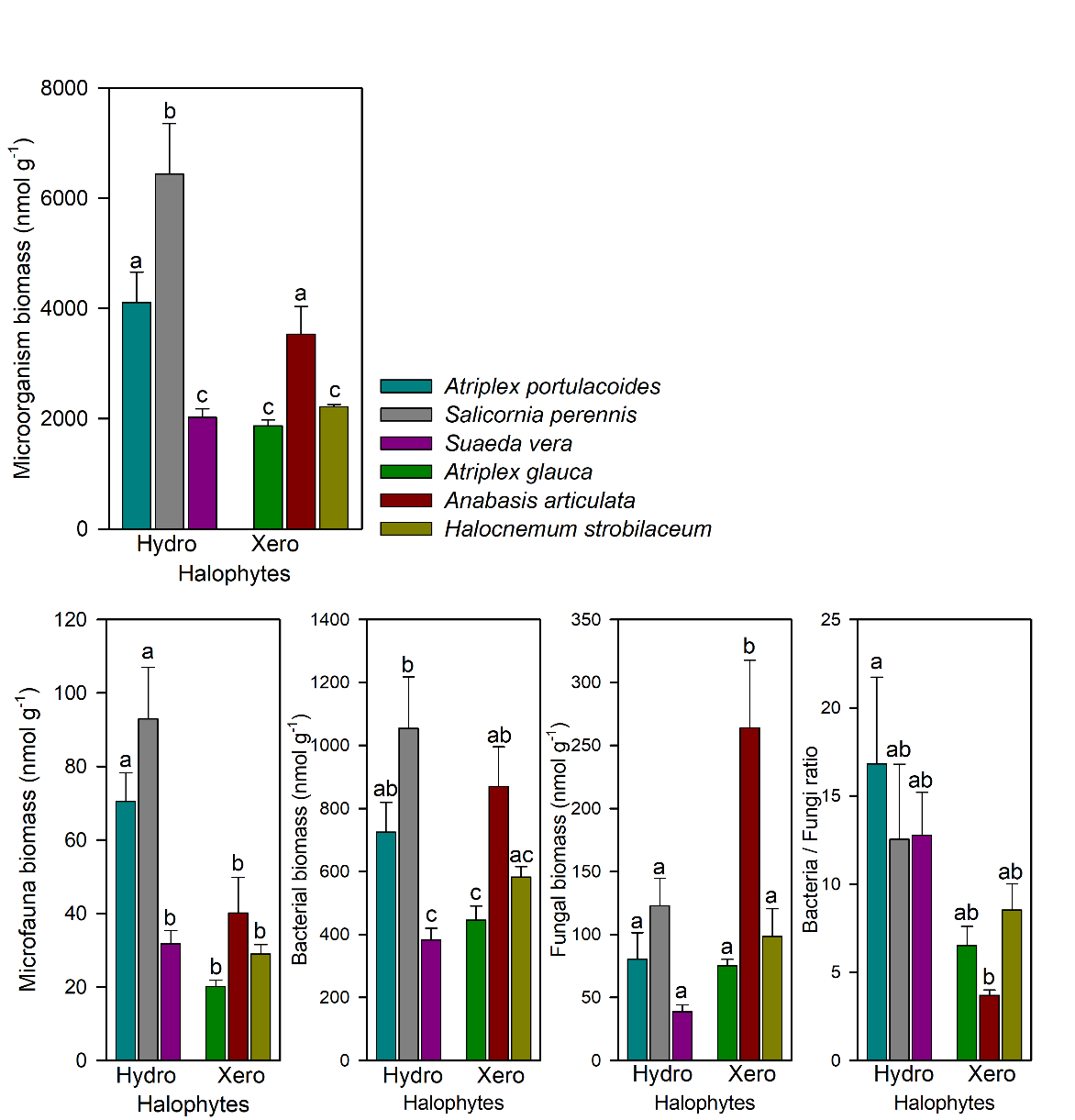
**Figure S3.** Microorganism, microfauna, bacterial, and fungal biomasses and bacteria/fungal ratio of sampled soils in the rhizosphere of hydrohalophyte and xerohalophyte species. Each value represents the mean of nine replicates (n = 3 per plot) ± SE. Different lowercase letters indicate means that are significantly different among species, based on ANOVA followed by Tukey’s HSD post-hoc test (p < 0.05).


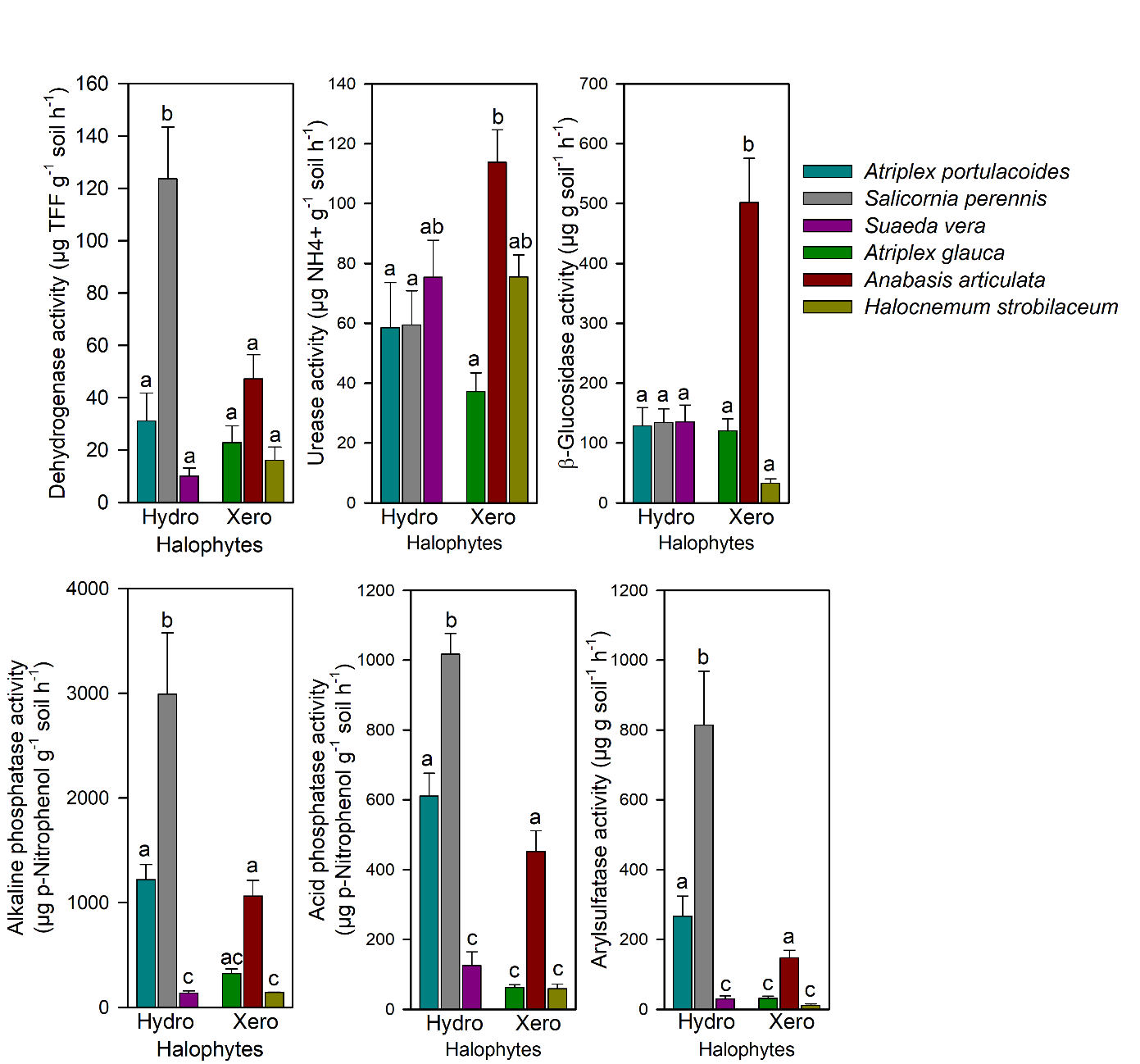


**Figure S4.** Dehydrogenase, urease, β-glucosidase, alkaline phosphatase, acid phosphatase, and arylsulfatase activities of sampled soils in the rhizosphere of hydrohalophyte and xerohalophyte species. Each value represents the mean of nine replicates (n = 3 per plot) ± SE. Different lowercase letters indicate means that are significantly different among species, based on ANOVA followed by Tukey’s HSD post-hoc test (p < 0.05).
